# Acquired pure red cell aplasia and T cell large granular lymphocytic leukaemia in patients with autoimmune polyglandular syndrome type 1

**DOI:** 10.1186/s12920-020-00866-y

**Published:** 2021-01-19

**Authors:** Jing Ruan, Xuan Wang, Xianyong Jiang, Miao Chen

**Affiliations:** Hematology Department, Peking Union Medical College Hospital, Peking Union Medical College and Chinese Academy of Medical Sciences, Beijing, 100730 China

**Keywords:** Pure red cell aplasia, Large granular lymphocytic leukaemia, Autoimmune polyendocrine syndrome type 1, AIRE gene mutation

## Abstract

**Background:**

Pure red cell aplasia (PRCA) and large granular lymphocytic leukaemia (LGLL) are very rare complications of autoimmune polyendocrine syndrome type 1 (APS1). Here, we report a case of APS1 with PRCA and LGLL. Previous cases were reviewed, and possible mechanisms are discussed.

**Case presentation:**

A 31-year-old female presented with anaemia and was diagnosed with PRCA in our centre. She also had hypoparathyroidism for 24 years, premature ovarian failure for 10 years, osteoporosis for 5 years, recurrent pneumonia with bronchiectasis for 4 years and chronic diarrhoea for 1 year. Boosted whole-exome analysis showed *AIRE* heterozygous mutations, confirming the diagnosis as APS1. LGLL was diagnosed during follow-up. The PRCA responded well to glucocorticoid. treatment

**Conclusion:**

*AIRE* is causally related to the development of LGLL and consequent PRCA, which may be due to some immunological mechanisms.

## Background

Pure red cell aplasia (PRCA) is a syndrome defined by anormocytic normochromic anaemia with severe reticulocytopaenia and marked reduction or absence of erythroid precursors from the bone marrow [1]. Acquired PRCA is an autoimmune disorder involving autoantibodies or T cell-mediated inhibition of erythropoiesis. Secondary acquired PRCA may be associated with lymphoproliferative disorders, such as T cell large granular lymphocytic leukaemia (T-LGLL) [2], which is a monoclonal disorder of CD8-positive suppressor T cells. The clone of LGLL may target an erythroid antigen, resulting in erythroid hypoplasia and anaemia [2].

Here, we report a patient who presented with PRCA and T-LGLL and was ultimately diagnosed with autoimmune polyglandular syndrome type 1 (APS-1). APS-1 is a rare autosomal recessive or autosomal dominant disorder caused by mutations in the autoimmune regulator (*AIRE*) gene that help to reinforce immune tolerance by preventing the maturation of autoreactive T cells. The estimated prevalence is approximately 1:80,000 in most countries [3]. Clinical manifestations of APS-1 include mucocutaneous candidiasis, hypoparathyroidism, adrenal insufficiency and other autoimmune diseases [3–5]. PRCA and/or LGLL are very rare complications of APS-1. Previous cases were reviewed, and possible immunological mechanisms are discussed.

## Case presentation

**Fig. 1:**
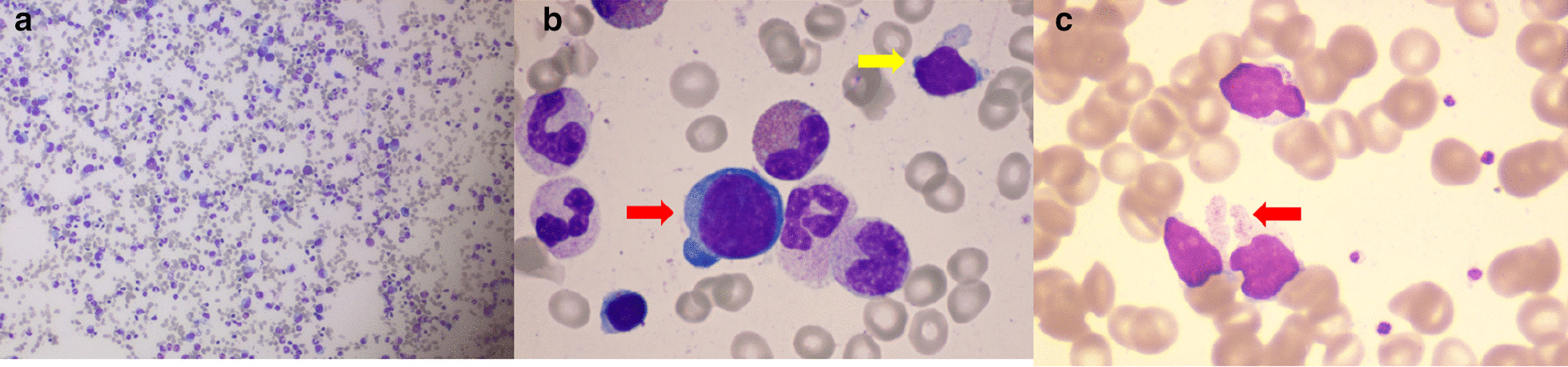
The bone marrow aspirate and the peripheral blood smear. **a** A bone marrow smear showed normocellular marrow with a myeloid erythroid ratio of 68:1 (original magnification, × 100); **b** A bone marrow smear showed a few basophilic erythroblasts (red arrow) without polychromatic and orthochromatic normoblasts, which indicated the diagnosis of PRCA. A few granular lymphocytes (yellow arrow) were also found in the bone marrow at that time (original magnification, × 1000). **c** A peripheral blood smear showed large granular lymphocyte (red arrow) proliferation (original magnification, × 1000)

A 31-year-old female presented with a pale face and tachycardia for 3 months. She was admitted in March 2018. Her past history included hypoparathyroidism for 24 years, premature ovarian failure for 10 years, osteoporosis for 5 years, recurrent pneumonia with bronchiectasis for 4 years and chronic diarrhoea for 1 year. Blood counts revealed severe anaemia (haemoglobin 50 g/L, mean corpuscular volume 99.5 fl) with normal white blood cells (WBCs) and platelets (WBCs 6.47 × 10^9^/L, neutrophils 2.45 × 10^9^/L, lymphocytes 3.6 × 10^9^/L, platelets 245 × 10^9^/L). The absolute reticulocyte count was 1.7 × 10^9^/L. No haemorrhage, haemolysis or nutritional anaemia was found. Intrinsic factor antibodies were positive (43.15 Au/ml). The glutamic acid decarboxylase antibody (GAD-Ab) level was 35 IU/ml. Anti-Ro 52 was positive (+++). A bone marrow smear showed normocellular marrow with a few basophilic erythroblasts (myeloid erythroid ratio 68:1), which indicated PRCA (Fig. 1). The ASXL1 mutation (NM_015338:exon12: c. C3946T: p.R1316C, variant allele fraction 51.28%) was detected, and her karyotype was normal. Flow cytometric analysis of the immunophenotype of the bone marrow showed that 96% of the lymphocytes were T cells, with an inversion ratio of CD4/CD8 cells. CD25, CD11c and CD56 were highly expressed, while CD16 was negative. Other secondary factors of PRCA were also evaluated. Virology testing, including for parvovirus B19, human immunodeficiency virus, Epstein-Barr virus and cytomegalovirus, yielded negative results. Rheumatoid factor, complement, and serum immunoglobulins were normal; antinuclear antibodies and antineutrophil cytoplasmic antibodies were negative. No solid tumour or lymphadenopathy was found on enhanced CT of the chest, abdomen or pelvis. Endocrine evaluation indicated adrenal insufficiency, with low adrenocorticotropic hormone levels. Along with her past medical history, autoimmune polyendocrine syndrome type 1, which can present with PRCA, was suspected. Boosted whole-exome analysis confirmed heterozygous mutations c.371C>T (p.Pro124Leu)(NM_000383.3) and c.623G>T (p.Gly208Val) (NM_000383.3) of the *AIRE* gene. Sanger’s sequencing showed that both mutations were inherited from her father, who had no symptoms, whereas the mother carried wild-type genes. In one report from the ClinVar database, c.371C>T is predicted to be likely pathogenic; c.623G>T is predicted to be damaging using PolyPhen and SIFT software.

**Fig. 2:**
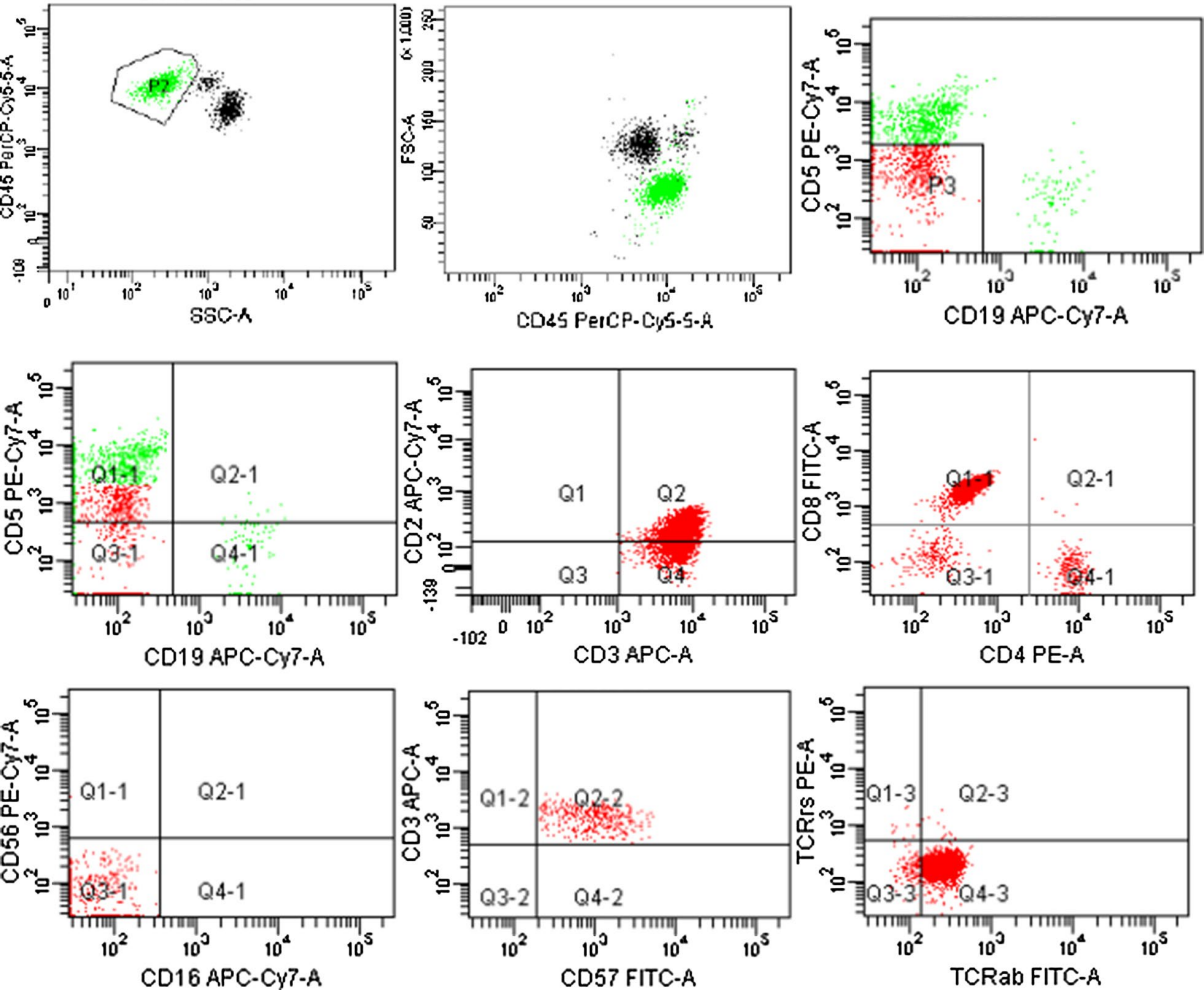
Immunophenotyping of peripheral blood samples. Lymphocytes (green colour, P2) were mainly T cell lymphocytes (92%). A total of 58.7% of the lymphocytes (red colour, P3) were found to express CD3, CD57 and TCRαβ without CD56

The patient did not respond to cyclosporine A (CsA) at first due to malabsorption caused by diarrhoea, though methylprednisolone (32 mg qd) efficiently increased haemoglobin to normal levels in 1 month. However, during follow-up, we noticed persistent lymphocytosis after 2 months. The peripheral blood smear revealed 60% lymphocytes, and among them, 70% were large granular lymphocytes (Fig. 1). Immunophenotyping confirmed CD3, CD57 and TCRαβ without CD56 expression (Fig. 2). T-cell receptor variable β-chain (TCRVβ) repertoire analysis showed that TCRVβ14 accounted for 89.63% of the CD7dim + CD5dim + cells, which were monoclonal TCRVβ cells. TCR gene rearrangement analysis revealed TCRγ. Thus, T large granular lymphocyte leukaemia (T-LGLL) was diagnosed.

**Fig. 3:**
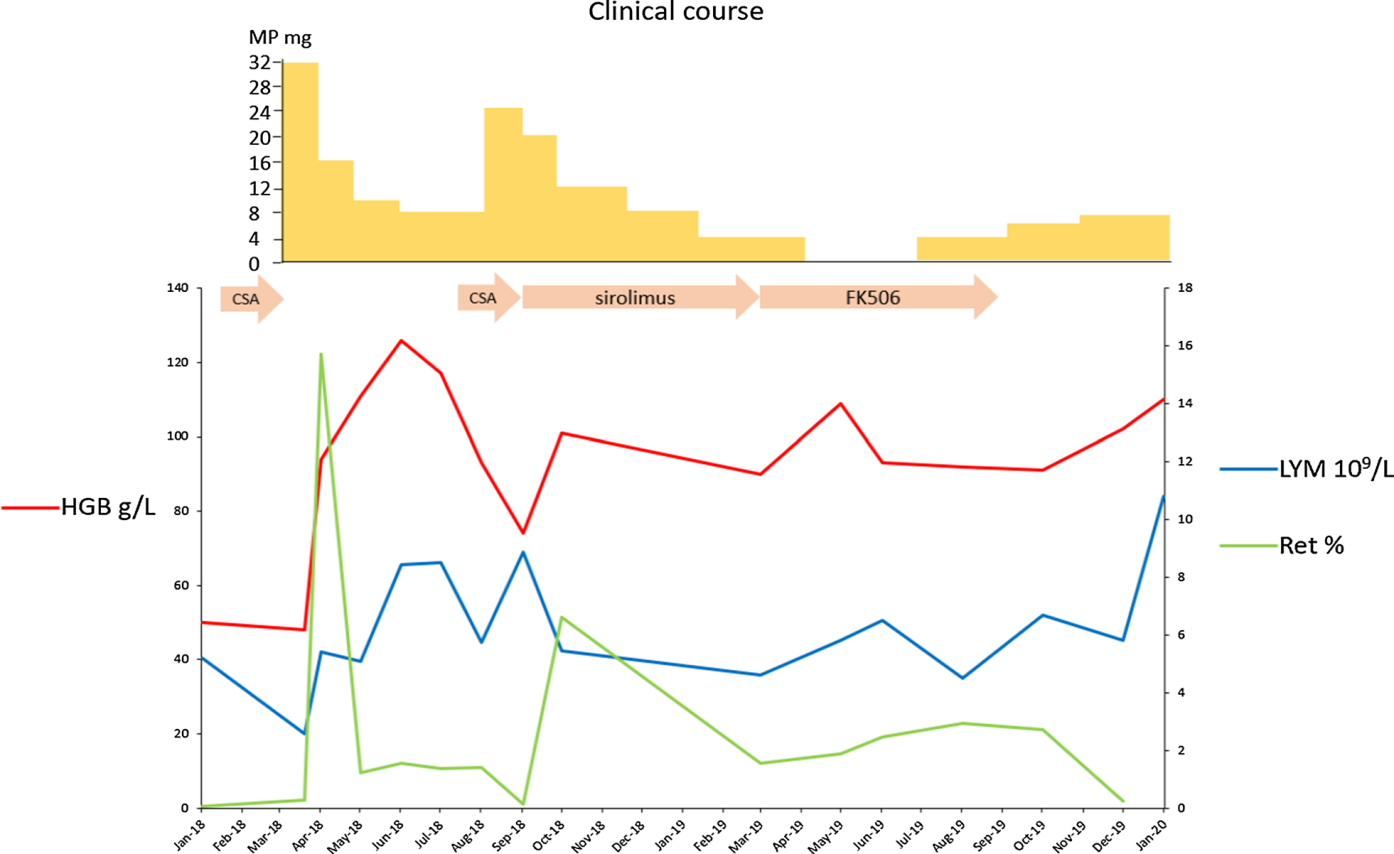
The clinical course of the patient

During the reduction of glucocorticoids, cyclosporine A was added again but caused acute renal infarction (Cr 415 µmol/L), which was reversed after cessation of cyclosporine A. Sirolimus or tacrolimus combined with low-dose glucocorticoids only maintained 90 g/L haemoglobin after 6 months. Recently, her haemoglobin level recovered to 110 g/L with methylprednisolone 8 mg/day, though lymphocytosis persisted (Fig. 3).

## Discussion and conclusions

Autoimmune polyglandular syndrome type 1 (APS-1) is caused by mutations in the AIRE gene, which is located on chromosome 21 (21q22.3) and encodes the AIRE protein. Here, we report an APS-1 patient who harboured heterozygous mutations in *AIRE,* including c.371C > T (p.Pro124Leu) and c.623G > T (p.Gly208Val). These two mutations are novel and likely pathogenic according to the ClinVar database and function-prediction software. AIRE functions as a transcription factor and regulates the transcription of peripheral tissue antigens in thymic medullary epithelial cells. It plays a key role in shaping central and peripheral immunological tolerance by facilitating negative selection of autoreactive T cells in the thymus and inducing a specific subset of regulatory T cells [6, 7].

In the absence of AIRE, autoimmunity develops from two failed tolerance mechanisms targeting more than one endocrine organ and a nonendocrine organ. APS-1 can be diagnosed clinically based on the appearance of at least two of the three conditions: candidiasis, hypoparathyroidism and adrenocortical failure [4, 5]. In this patient, hypoparathyroidism was the first manifestation of APS-1 and occurred at the age of seven years old. She also had adrenal insufficiency and other endocrinological symptoms, such as premature ovarian and osteoporosis. Mucocutaneous candidiasis was not observed. Moreover, she had chronic diarrhoea and recurrent pneumonia with bronchiectasis.

Haematological abnormalities have also been identified in APS-1. In the largest cohort study of APS-1, consisting of 112 patients in Russia, pernicious anaemia was seen in 8% (10/112) [8]. Pernicious anaemia is the most common cause of anaemia as a result of autoimmune gastritis with vitamin B12 deficiency [3]. In the above study, PRCA was only found in 1% of the patients [8], with coexistence with large granular lymphocyte leukaemia in some. Autoimmune haemolytic anaemia is very rare [9, 10].

Eight cases of PRCA associated with APS-1 have been reported [11–17], of which only four had coexisting LGLL [15–17]. All four patients were female, and their first manifestations of APS-1 were during childhood. PRCA and T-LGLL were diagnosed simultaneously. Three patients were in their 20 s, and one was 46 years old. In the current case, APS-1 preceded PRCA and T-LGLL by 24 years. This may not be a coincidence, as APS-1, PRCA and T-LGLL are all rare diseases, and PRCA and LGLL can even develop in siblings with the same *AIRE* mutation [18]. To date, some mechanisms for patients diagnosed with LGLL with PRCA have been explored [19]. Regarding molecular mechanisms, STAT3 mutations have been found in some PRCA patients with LGLL [20], though it remains uncertain whether STAT3-mutated T cells have an inhibitory effect on erythroid cell production. Furthermore, STAT5b mutations have essential roles in the survival and proliferation of haematopoietic cells. Nonetheless, the present patient did not carry STAT3 or STAT5b mutations. Regarding the immune mechanism, Handgretinger et al. [21] reported that in a patient with γδT-LGLL-associated PRCA, γδT-LGLs inhibited erythroid precursors by KIRs. It is possible that autoimmunity of APS-1 may predispose an individual to the development of clonal proliferation of LGLs. The T-LGLL clone might recognize a self-antigen and expand due to AIRE regulatory failure and lack of deletion of that T cell clone. The LGLL clone targets an erythroid antigen, resulting in PRCA.

Treatment responses to glucocorticoid and immunosuppressive drugs such as cyclophosphamide [12, 15] or mycophenolate-mofetil [13] vary among cases. In our case, glucocorticoids resulted in remission of PRCA, though lymphocytosis persisted. Sirolimus and tacrolimus failed to maintain normal haemoglobin levels.

In conclusion, we report a rare case of APS1 caused by *AIRE* mutations presented with PRCA and LGLL. With further literature review, *AIRE* is thought to be causally related to the development of LGLL and consequent PRCA due to some immunological mechanisms which need further investigation.

## Data Availability

The datasets generated and/or analysed during the current study are available in the NCBI BioProject database under the accession number PRJNA674510.

## Abbreviations

PRCA: Pure red cell aplasia
LGLL: Large granular lymphocytic leukaemia
APS1: Autoimmune polyendocrine syndrome type 1
AIRE: Autoimmune regulator
WBC: White blood cell
GAD-Ab: Glutamic acid decarboxylase antibody
ASXL1: Additional sex combs like 1, transcriptional regulator
CsA: Cyclosporine A
TCR: T cell receptor
